# Research on Establishing Corneal Edema after Phacoemulsification Prediction Model Based on Variable Selection with Copula Entropy

**DOI:** 10.3390/jcm12041290

**Published:** 2023-02-06

**Authors:** Yu Luo, Guangcan Xu, Hongyu Li, Tianju Ma, Zi Ye, Zhaohui Li

**Affiliations:** 1 Medical School of Chinese People’s Liberation Army, No. 28 Fuxing Road, Haidian District, Beijing 100853, China; 2 Department of Ophthalmology, Chinese People’s Liberation Army General Hospital, No. 28 Fuxing Road, Haidian District, Beijing 100853, China

**Keywords:** corneal edema, phacoemulsification, nomogram, prediction model, copula entropy

## Abstract

Background: Corneal edema (CE) affects the outcome of phacoemulsification. Effective ways to predict the CE after phacoemulsification are needed. Methods: On the basis of data from patients conforming to the protocol of the AGSPC trial, 17 variables were selected to predict CE after phacoemulsification by constructing a CE nomogram through multivariate logistic regression, which was improved via variable selection with copula entropy. The prediction models were evaluated using predictive accuracy, the area under the receiver operating characteristic curve (AUC), and decision curve analysis (DCA). Results: Data from 178 patients were used to construct prediction models. After copula entropy variable selection, which shifted the variables used for prediction in the CE nomogram from diabetes, best corrected visual acuity (BCVA), lens thickness and cumulative dissipated energy (CDE) to CDE and BCVA in the Copula nomogram, there was no significant change in predictive accuracy (0.9039 vs. 0.9098). There was also no significant difference in AUCs between the CE nomogram and the Copula nomogram (0.9637, 95% CI 0.9329–0.9946 vs. 0.9512, 95% CI 0.9075–0.9949; *p* = 0.2221). DCA suggested that the Copula nomogram has clinical application. Conclusions: This study obtained a nomogram with good performance to predict CE after phacoemulsification, and showed the improvement of copula entropy for nomogram models.

## 1. Introduction

Phacoemulsification is the procedure of choice for the treatment of cataracts, with over 20 million phacoemulsification performed worldwide each year [1,2]. Corneal edema (CE) is a common complication after phacoemulsification, but it sometimes does not receive enough attention from clinicians. In fact, an edematous cornea can affect a patient’s vision, cause discomfort such as eye pain and photophobia, and even increase anxiety [3,4]. Critically, CE occurring on the first day after phacoemulsification is strongly correlated with early (3 months) postoperative corneal endothelial cell density (ECD), and is an independent risk factor for long-term (10 years) postoperative corneal endothelial cell loss [5,6]. Corneal endothelial cells are not renewable, and their loss greatly increases the risk of corneal endothelial decompensation [4,7].

CE affects the visual outcome of phacoemulsification and is essential for aggressively preventing postoperative CE. Although there are studies analyzing the risk factors for the development of postoperative CE [3,7,8], accurate predictors are still lacking, hindering effectively preventing CE after phacoemulsification.

Hence, there is a necessity to develop a reliable predictive model for assessing the possibility of CE occurring immediately after phacoemulsification to help clinicians in performing timely interventions. For the above purpose, we constructed a nomogram of CE after phacoemulsification as a prediction model on the basis of patients’ clinical characteristics and surgical data. A study showed the advantages of copula entropy compared to other statistical methods for variable selection [9]. We used the method of calculating the copula entropy of each potential predictor in the process of constructing the model for variable selection, and evaluated the effect of copula entropy on the improvement of the prediction model. In addition, several recent studies showed that prediction models constructed with machine learning methods could achieve good predictive performance for some clinical outcomes in patients after cataract surgery [10,11,12,13]. As a comparison for the nomogram of CE, we chose random forest, a representative algorithm of machine learning, to construct another CE prediction model, and evaluated the improvement of copula entropy on the random forest model.

## 2. Materials and Methods

### 2.1. Data

This is a secondary analysis of data from the active-fluidics versus gravity-fluidics system in phacoemulsification for age-related cataract (AGSPC) trial, a completed prospective, randomized, double-masked, controlled clinical study that focused on the effects of the active-fluidics system (AFS) compared to the gravity-fluidics system (GFS) in phacoemulsification for age-related cataracts [14]. A total of 178 patients were included in the construction of a prediction model for CE. Of the 178 patients, 107 came from the AGSPC trial, 53 of whom used AFS and 54 used GFS for phacoemulsification. The other 71 patients eligible for the AGSPC protocol included patients who had been lost to follow-up at 1 week, 1 month, or 3 months after the surgery, patients who had specified the use of AFS or GFS, and patients who had consented to data collection, but declined to participate in the AGSPC trial. All patients included in this study completed at least one day of follow-up. Their demographics, perioperative examination, and surgery-related data were collected and are shown in Supplementary Table S1. Regardless of whether they were included in the AGSPC trial, all subjects had given their informed consent for inclusion before they participated in this study. The study was conducted in accordance with the Declaration of Helsinki, and the protocol was approved by the Ethics Committee of the Chinese People’s Liberation Army (PLA) General Hospital (no. S2021-068-01) [14,15]. All patients in the prediction model were recruited from March 2021 to March 2022, and were diagnosed with age-related cataracts and underwent phacoemulsification at the Chinese PLA General Hospital in Beijing, China. More specific details of the AGSPC protocol, including patient inclusion and exclusion criteria, perioperative examination items, and data collection, can be seen in the published studies [14,15].

### 2.2. Clinical Outcome and Predictors

The diagnosis of age-related cataracts and the grading of lens nuclear hardness were determined by the same senior ophthalmologist under a slit lamp according to the Lens Opacities Classification System II criteria [16]. An experienced ophthalmologist performed all the operations with the same procedure and an Intrepid balanced tip in the CENTURION^®^ Vision System (Alcon Laboratories, Fort Worth, TX, USA). When the phacoemulsification was performed with the AFS, the target IOP was set at 50 mmHg. When the surgery was performed with the GFS, the bottle height was put at 90 cm. The vacuum level and aspiration flow rate were 450 mmHg and 45 cc/min, respectively, in both systems. Patients who participated in the AGSPC were grouped on the basis of the randomization results. Patients who did not agree to participate in the clinical trial, but were eligible for AGSPC, chose the surgical system according to their own desires. All patients were treated with the same prescription and viscoelastics (medical sodium hyaluronate gel, Iviz, Bausch + Lomb, Rochester, NY, USA) in the perioperative period. More details could be extracted from the protocol [15].

The prediction model was to predict the incidence of CE in patients on the first day after phacoemulsification. One ophthalmologist completed slit-lamp biomicroscopic and corneal endothelial microscopic examinations for all patients at one day postoperatively. Patients were asked about any discomfort at the same time. When the corneal stroma shows diffuse gray or milky clouding, CE is defined. However, if the opacity of the cornea is limited to the vicinity of the surgical incision and does not reach the visual axis area, the cornea is not classified as edema. Central corneal thickness (CCT) measured with a noncontact specular microscope (SP·3000P, Topcon, Tokyo, Japan) is another auxiliary indicator to assess CE. When CE is detected under a slit lamp, there is a notable increase in CCT compared to the preoperative period, which is usually greater than 50 μm. Nevertheless, there was a subset of patients who did not have obvious diffuse corneal clouding despite a significant increase in CCT. This may have correlated with the patient’s different preoperative CCT, so observation under a slit lamp was adopted as the main method of diagnosing CE. Some patients postoperatively developed Descemet’s membrane striae, but did not show a significant CCT increase and opacity in the visual axis area. Therefore, these patients were considered to have only mild corneal complications that were not defined as CE.

We reviewed previously published studies that focused on risk factors for the development of CE after phacoemulsification, and referenced potential risk factors therein [5,17,18,19]. Combining the data collected under the AGSPC protocol, we identified 17 potential predictors to construct prediction models for CE after phacoemulsification: gender, age, hypertension (HBP), diabetes (Dia), best corrected visual acuity (BCVA), preoperative intraocular pressure (Pre IOP), nuclear hardness (NH), axial length (AL), anterior chamber depth (ACD), lens thickness (LT), CCT, ECD, fluidics, cumulative dissipated energy (CDE), ultrasound time (U/S time), estimated fluid used (EFU), and total aspiration time (TAT).

### 2.3. Model Development

First, multivariate logistic regression was performed on the basis of the 17 selected variables with CE on the first postoperative day, and a CE nomogram was formed on the basis of the results of the multivariate logistic regression. Second, to assess the effect of variable selection via copula entropy on the improvement of the CE nomogram, we calculated the copula entropy between each variable, and CE with the reported method of Ma [9]; we ranked the variables according to the copula entropies. We selected eight variables larger than the median of all copula entropies, performed another multivariate logistic regression with CE on the first postoperative day, and constructed a Copula nomogram on the basis of the regression results. Considering that all cataract patients included in the construction of models were from the same medical center, we did not divide the source of data into training and testing sets. Instead, we used the leave-one-out (LOO) method for the validation of the two nomograms, and the final output showed the predictive accuracy. In the comparison, the best-performing nomogram was selected as the final adopted model for predicting the risk of CE on the first day after phacoemulsification.

In addition, we constructed a random forest (RF) model to predict CE on the first day after phacoemulsification via the 17 selected variables. According to the ranking of the copula entropy of each variable, the variables with the smallest copula entropy were sequentially removed from the total variables incorporated into the random forest model, and the remaining variables were used to construct new random forest models. The final output was the predictive accuracy and out-of-bag error (OOB) rate of all random forest models, and we chose the result with the best and stablest prediction performance as the Copula random forest model (Copula RF). We also calculated the mean decreased Gini of the variables in each random forest model, and separately ranked the mean decreased Gini values of each random forest model.

### 2.4. Model Evaluation

The main evaluation criteria for the prediction model were the predictive accuracy and the receiver operating characteristic (ROC) curve-based area under the ROC curve (AUC) [20]. We plotted ROC curves and calculated AUCs for a CE nomogram, Copula nomogram, RF, and Copula RF, and performed statistical comparisons among these AUCs. Furthermore, we plotted calibration curves to assess the agreement between predictions and observations in different percentiles of the predicted values, preventing nomograms with good predictive performance from overfitting and becoming unusable. To evaluate the clinical application value of the prediction models, we conducted decision curve analysis (DCA) by separately calculating the net benefit of each prediction model at different threshold probabilities [21]. The DCA curves above the straight line, representing a net benefit of zero, show that the prediction models have clinical application value.

### 2.5. Statistics

All statistical analyses were conducted with R software 4.1.3 (R Foundation for Statistical Computing, Vienna, Austria). Copula entropy was calculated using the “copent” package, and other packages were “rms”, “readr”, “forestplot”, “copent”, “caret”, “randomForest”, “MASS”, “dcurves”, and “pROC”. The continuous variables are listed as the mean (minimum, maximum) and median (interquartile range). The categorical variables are listed as numbers and percentages. Multivariate logistic regression was used to analyze the potential predictors, and the results were used to construct the nomograms. The logistic regression results are presented with the odds ratio (OR), their 95% confidence intervals (CI 95%), and *p*-values. We used a bootstrapping method of sampling 1000 times for the calibration curves of the nomograms. The AUCs between the different prediction models were compared using the Z-test method, and the AUCs with 95% CI for each prediction model are also presented. All *p* values < 0.05 in the study were considered significant.

## 3. Results

### 3.1. Clinical Outcomes and Predictors

CE had occurred in 30 of 178 patients (16.85%) at 1 day after phacoemulsification. Table S1 shows detailed information on the 17 variables and the CE. There were no missing values in the data for constructing the prediction model.

### 3.2. Model Development

The results of the multivariate logistic regression between the 17 potential predictors and CE are displayed in Figure S1A. Independent risk factors affecting the development of CE were Dia (OR 28.09; 95% CI, 1.66–474.1; *p* = 0.0207), BCVA (OR 13.30; 95% CI, 1.69–104.44; *p* = 0.0139), LT (OR 0.05; 95% CI, 0.01–0.49; *p* = 0.0096), and CDE (OR 20,405; 95% CI, 9.41–44,268,000; *p* = 0.0113). Independent risk factors were used to construct the CE nomogram (Figure S1B). The CE nomogram was validated with the LOO method, resulting in a predictive accuracy of 90.39%.

**Figure 1:**
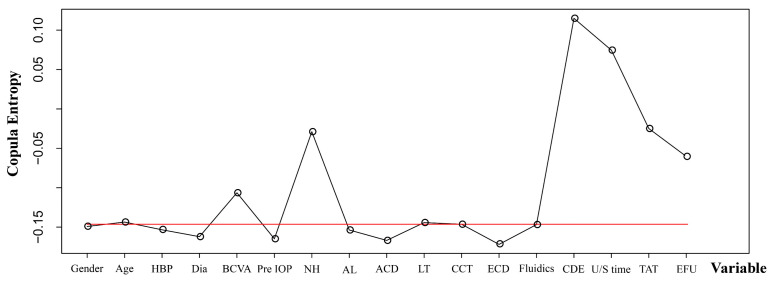
Copula entropies of 17 selected potential predictors. The red line represents the median of all copula entropies. ACD: anterior chamber depth; AL: axial length; BCVA: best corrected visual acuity; CCT: central corneal thickness; CDE: cumulative dissipated energy; Dia: diabetes; ECD: endothelial cell density; EFU: estimated fluid used; HBP: hypertension; LT: lens thickness; NH: nuclear hardness; Pre IOP: preoperative intraocular pressure; TAT: total aspiration time, U/S time: ultrasound time.

**Figure 2:**
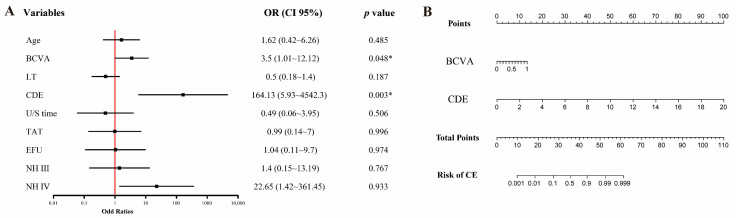
(**A**): Multivariate logistic regression model for predicting CE after phacoemulsification with the variables selected via copula entropy. (**B**) Copula nomogram in patients with CE after phacoemulsification. *: The *p* value is less than 0.05. BCVA: best corrected visual acuity; CDE: cumulative dissipated energy; EFU: estimated fluid used; LT: lens thickness; NH: nuclear hardness; TAT: total aspiration time, U/S time: ultrasound time.

The copula entropy between the 17 potential predictors and CE is displayed in Figure 1. The eight variables greater than the median of all copula entropies were CDE, U/S time, TAT, NH, EFU, BCVA, age, and LT, in descending order. The results of the multivariate logistic regression between these selected variables and CE are displayed in Figure 2A. Variables significantly related to CE on the first postoperative day were BCVA (OR 3.5; 95% CI, 1.01–12.12; *p* = 0.048) and CDE (OR 164.13; 95% CI, 5.93–4542.3; *p* = 0.003). These results were constructed as the Copula nomogram (Figure 2B). The Copula nomogram was validated with the LOO method, resulting in a predictive accuracy of 90.98%.

**Figure 3:**
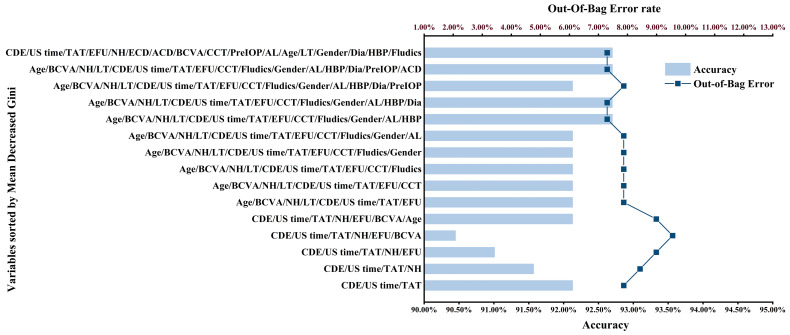
The predictive accuracy and out-of-bag error rate of each random forest model generated after selecting variables on the basis of copula entropy. ACD: anterior chamber depth; AL: axial length; BCVA: best corrected visual acuity; CCT: central corneal thickness; CDE: cumulative dissipated energy; Dia: diabetes; ECD: endothelial cell density; EFU: estimated fluid used; HBP: hypertension; LT: lens thickness; NH: nuclear hardness; Pre IOP: preoperative intraocular pressure; TAT: total aspiration time, U/S time: ultrasound time.

The mean decreased Gini values and ranking of each variable in the RF model incorporating all variables are shown in Figure S2. The mean decreased Gini could only represent the degree of contribution of each variable in the random forest model. Figure 3 shows the predictive accuracy and OOB rate of each random forest model generated after variable selection based on copula entropy. The y axis of Figure 3 indicates the mean decreased Gini ranking of the remaining variables after the selection of each copula entropy variable, where ECD was the variable with the smallest copula entropy, and the mean decreased Gini ranking in each random forest model after excluding ECD tended to be stable. Combining the maximal predictive accuracy (92.7%) and the minimal OOB rate (7.3%), the random forest model after excluding ECD was selected as the Copula RF.

### 3.3. Model Evaluation

**Figure 4:**
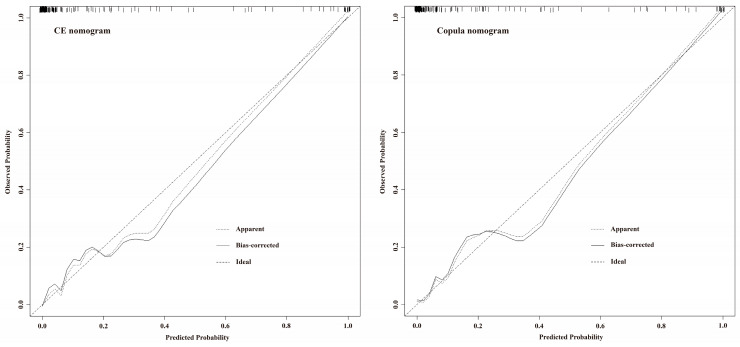
The calibration curves for the CE and copula nomograms.

**Figure 5:**
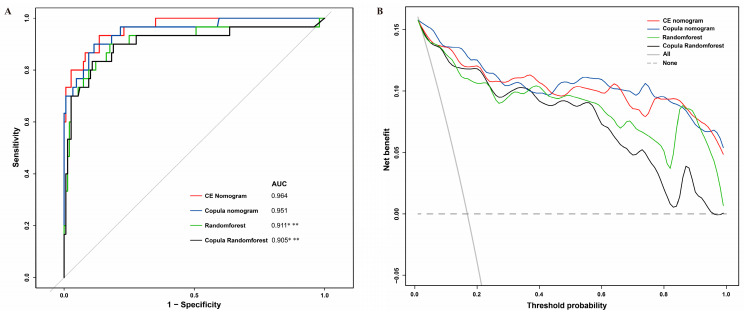
(**A**): ROC curves and AUCs for all prediction models. (**B**): DCA for all prediction models. ROC: receiver operating characteristic; AUC: area under ROC curve; DCA: decision curve analysis; *: AUCs were significantly different from the CE nomogram; **: AUCs were significantly different from the Copula nomogram.

The calibration curves for both the CE nomogram and the Copula nomogram reveal good predictive accuracy between the actual probability and predicted probability (Figure 4). The ROC curves of the CE nomogram, Copula nomogram, RF, and Copula RF are shown in Figure 5A. The AUC was 0.9637 (95% CI, 0.9329–0.9946) for the CE nomogram, 0.9512 (95% CI, 0.9075–0.9949) for the Copula nomogram, 0.9113 (95% CI, 0.8382–0.9843) for the RF, and 0.9046 (95% CI, 0.8275–0.9817) for the Copula RF. There was no significant difference between the AUCs of the CE nomogram and Copula nomogram (*p* = 0.2221), but they were significantly greater than the AUCs of the RF and Copula RF (Figure 5A). The DCA curves of all prediction models showed a net benefit in predicting postoperative CE at almost any threshold probability, suggesting good clinical application (Figure 5B).

## 4. Discussion

CE is a common complication after phacoemulsification. It not only impairs vision recovery and causes subjective discomfort to the patient, but can also interfere with the ophthalmologist’s judgment of the patient’s status of recovery by affecting the accuracy of IOP measurements [17]. In addition, CE had a significant effect on the loss of corneal endothelial cells after phacoemulsification [5,6]. In previous studies, concurrent diabetes, the vertical radius of the corneal curvature (diopters), the time of phacoemulsification, and surgical experience were considered important factors influencing the development of CE [17,19], but they were not sufficient to help the ophthalmologist in quickly and accurately determining the risk of CE after phacoemulsification.

On the basis of a balanced set of data, we established a well-performing nomogram for predicting the occurrence of CE after phacoemulsification using copula entropy. In addition, we used this method of constructing prediction models for the first time on an ophthalmology dataset that could provide an important reference for other ophthalmic researchers. The theory of copula entropy was first proposed by Ma et al. in 2008 on the basis of the definition of the copula density function, and confirmed the advantage of copula entropy over other statistical methods in variable selection with the UCI cardiology dataset in a 2019 study [9,22]. In our study, variable selection via copula entropy transformed the CE nomogram to predict with 4 variables into a Copula nomogram with 2 variables, reducing the complexity of the CE nomogram, and increasing the clinical utility of the prediction model without reducing the predictive accuracy. This indicates that copula entropy performs effective variable selection in the construction of nomograms.

Random forest is a representative algorithm of machine learning. The mean decreased Gini generated by the random forest is considered by some researchers useful for assessing the importance of variables and thus for the variable selection process [23,24,25], which is true for specific variables; for example, in this study, both the mean decreased Gini and the copula entropy results show that CDE is the most important variable influencing the occurrence of CE. However, in fact, the mean decreased Gini only represents the contribution of the variable within the random forest, but cannot determine the strength of the association between the variable and the outcome [26]. Reviewing the properties of our data shows that there were a few outliers within the ECD series, which may have been due to the exclusion of severe ocular disease in the patient inclusion. In the copula entropy ranking, ECD was the least related variable to CE, a consistent result with the clinical interpretability of the data. Interestingly, the ECD was ranked sixth in the ranking of mean decreased Gini. In the process of training the model, the occurrence of CE in patients with normal ECD as the wrong training subject would become noisy data for the random forest model, thus affecting the predictive accuracy and possibly leading to overfitting. The results of the copula entropy helped in analyzing and excluding the ECD, which is noisy data, and in determining the Copula RF without degrading the predictive performance.

Among the two ways of constructing prediction models, namely, nomograms and machine learning methods represented by RF, we prefer nomograms as a result of clinical use. Predictive performance is only one of the reasons. On the other hand, machine learning methods such as random forest can obtain prediction models with good performance by processing a large amount of input data, but this good performance is based on using much clinical information as the predictor. However, the clinical information of an individual in a patient population is often inadequate for using a specific machine learning prediction model due to the heterogeneity of clinical care. Although the number of predictors required by a random forest classifier can be reduced with the help of copula-based methods, this reduces the predictive accuracy, and affects the specificity and sensitivity of the prediction model [27]. The nomogram improved by copula entropy can minimize the clinical information required while maintaining good performance, and are more suitable for rapid determination of the risk of CE after phacoemulsification and other similar clinical scenarios.

CDE is the most important factor in the composition of a Copula nomogram, which correlates with the skill and experience of the surgeon. We found differences in CDE between AGSPC and other studies, resulting in diverse evaluations of the AFS [14,28,29,30]. The variation in different studies led us to use single-center data in this preliminary study with caution. This also implies that the Copula nomogram based on the CDE series in the AGSPC is likely useful only for experienced surgeons with similar surgical skills, which may affect the generalizability of the Copula monogram. The lack of validation of the nomograms using clinical data from other medical centers is the greatest limitation of this study. Nevertheless, the improved help of copula entropy in building prediction models is demonstrated in our results, and this method of building prediction models may provide insight to other researchers.

## 5. Conclusions

In summary, our analysis found that CDE and preoperative BCVA are significant risk factors for CE after phacoemulsification, and this resulted in a well-produced nomogram to predict CE after phacoemulsification. In addition, we introduced variable selection according to the copula entropy of each variable in the prediction model construction that improved the performance of the predictive model and confirmed that this method is worth being used in ophthalmic datasets.

## 6. Patents

This prediction model is protected by a Chinese patent (State Intellectual Property Office of China, 202211462005.7).

## Data Availability

The data presented in this study are available on request from the corresponding author.

## Supplementary Materials

The following supporting information can be downloaded at: https://www.mdpi.com/article/10.3390/jcm12041290/s1, Figure S1: (A) Multivariate logistic regression model for predicting CE after phacoemulsification with all variables. (B) CE nomogram in patients with CE after phacoemulsification; Figure S2: Mean decreased Gini of all variables and ranking in the random forest model; Table S1: Clinical information of the 17 potential predictors and the CE.
