# Supplementary material for: Research on Establishing Corneal Edema after Phacoemulsification Prediction Model Based on Variable Selection with Copula Entropy
Source: J Clin Med. 2023 Feb 6;12(4):1290. doi: 10.3390/jcm12041290 (PMC9963919; doi:10.3390/jcm12041290)
Supplement: Supplementary file 1 [file jcm-12-01290-s001.zip › Supplementary Table S1.pdf]

Supplementary Table S1. Clinical Information of 17 Potential Predictors and the Corneal Edema.

	**Mean (min, max)/ Number**	**Median (IQR)**
**Age** (y)	70.57(38,91)	73(14)
**BCVA**	0.4549(0.1,1)	0.5(0.3)
**Pre IOP** (mmHg)	13.87(7.4,23.4)	13.90(3.37)
**AL** (mm)	23.93(21.1,30.56)	23.62(1.61)
**ACD** (mm)	3.036(2.25,4.04)	3(0.44)
**LT** (mm)	4.621(3.34,5.63)	4.65(0.518)
**CCT** (µm)	511.8(429,621)	514(44.5)
**ECD** (/mm^2)	2556(1815,3409)	2574(486)
**CDE** (%S)	3.701(0,19.74)	2.85(3.083)
**U/S time** (s)	23.44(0,93.1)	18.55(16.06)
**TAT** (s)	151.3(55,284)	146.5(53.8)
**EFU** (cc)	47.09(20,95)	46(38,55)
**Gender**		
Male	77	43.26%
Female	101	56.74%
**HBP**		
Yes	83	46.63%
No	95	53.37%
**Dia**		
Yes	51	28.65%
No	127	71.35%
**NH**		
2	84	47.19%
3	86	48.31%
4	8	4.49%
**Fluidics System**		
Active	58	32.58%
Gravity	120	67.42%
**Corneal Edema**		
Yes	30	16.85%
No	148	83.15%

ACD, anterior chamber depth; AL, axial length; BCVA, best corrected visual acuity; CCT, central corneal thickness; CDE, cumulative dissipated energy; CE, corneal edema; Dia, diabetes; ECD, corneal endothelial cell density; EFU, estimated fluid used; HBP, hypertension; LT, lens thickness; NH, nuclear hardness; Pre IOP, preoperative intraocular pressure; TAT, total aspiration time; U/S time, ultrasound time.
