# Supplementary figures and images for: Research on Establishing Corneal Edema after Phacoemulsification Prediction Model Based on Variable Selection with Copula Entropy
Source: J Clin Med. 2023 Feb 6;12(4):1290. doi: 10.3390/jcm12041290 (PMC9963919; doi:10.3390/jcm12041290)

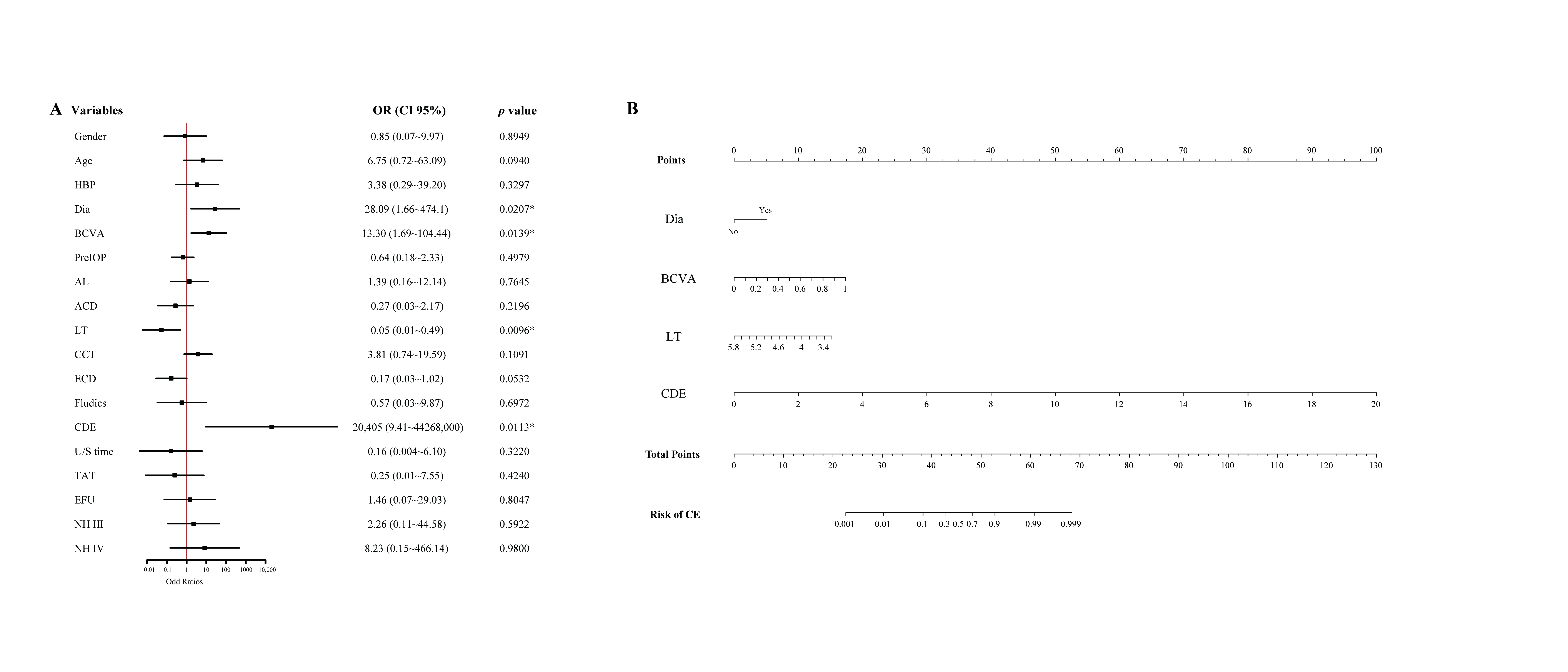

Supplement: Supplementary file 1 [file jcm-12-01290-s001.zip › Supplementary Figure 1.tif]

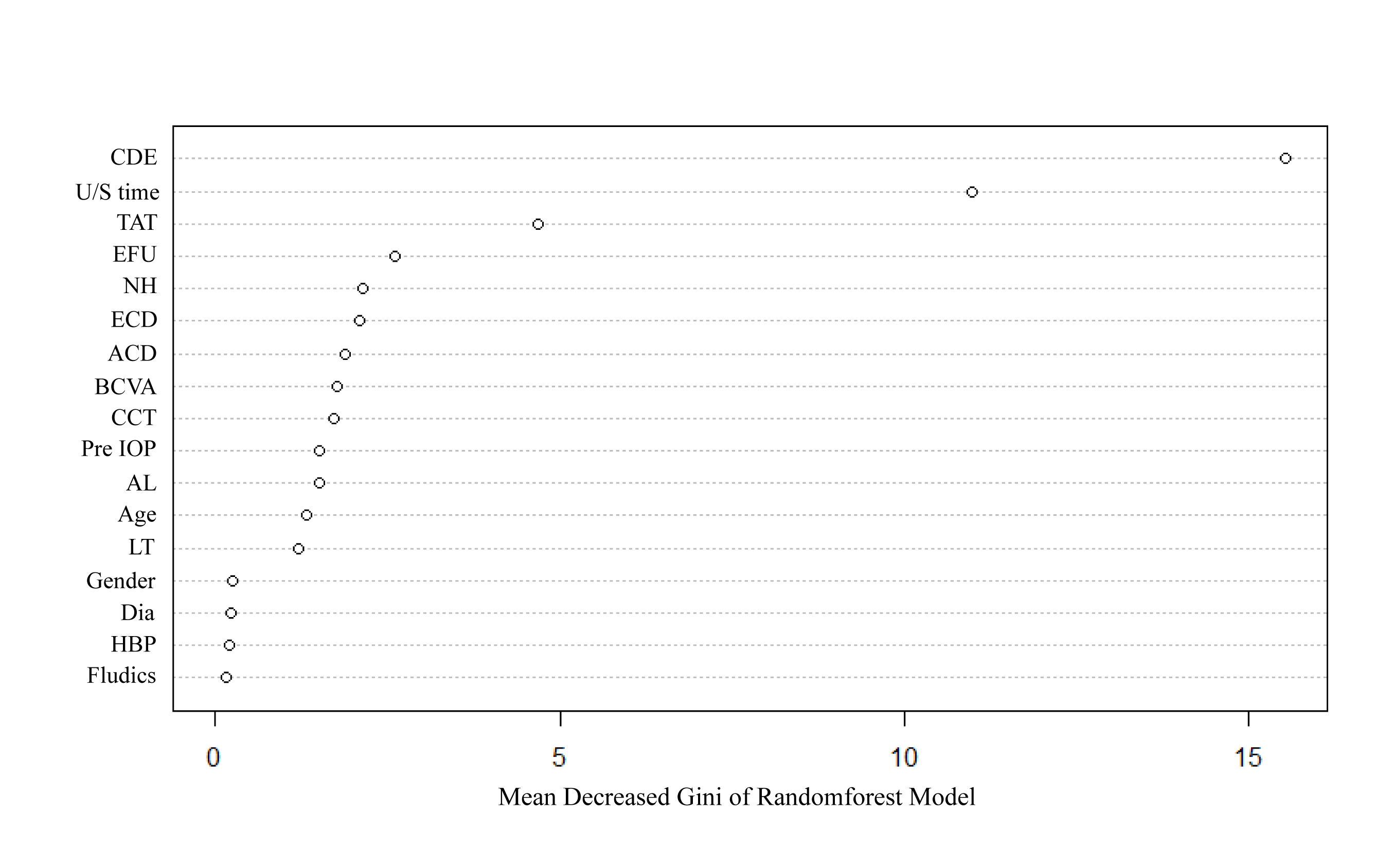

Supplement: Supplementary file 1 [file jcm-12-01290-s001.zip › Supplementary Figure 2.tif]
